# Does a mindfulness-augmented version of the German Strengthening Families Program reduce substance use in adolescents? Study protocol for a randomized controlled trial

**DOI:** 10.1186/s13063-020-4065-1

**Published:** 2020-01-28

**Authors:** Nicolas Arnaud, Christiane Baldus, Léa Josette Laurenz, Sonja Bröning, Maja Brandt, Sabrina Kunze, Maria Austermann, Linda Zimmermann, Anne Daubmann, Rainer Thomasius, the IMAC-Mind Consortium, Frauke Nees, Maren Prignitz, Stella Guldner, Herta Flor, Peter-Michael Sack, Karl Wegscheider, Antonia Zapf, Petra Meibert, Johannes Michalak, Virginia Molgaard, Kathy Hockaday, Lindsey Coombs

**Affiliations:** 1 German Centre for Addiction Research in Childhood and Adolescence, University Medical Centre Hamburg–Eppendorf, Hamburg, Germany; 2 Institute of Research and Education GmbH associated with the Medical School Hamburg (MSH), Hamburg, Germany; 3 Institute of Medical Biometry and Epidemiology, University Medical Centre Hamburg–Eppendorf, Hamburg, Germany

**Keywords:** Adolescence, Family, Mindfulness, Substance use, Self-regulation, Prevention

## Abstract

**Background:**

Mindfulness training (MT) for parents of adolescents has been shown to improve mental health and stress-related outcomes in individuals and their families. Studies of MT among young people are mainly delivered in educational or clinical settings, and there is a need for controlled studies on both parent-directed and adolescent-directed approaches. It is unclear whether MT has preventive effects for substance use outcomes. The primary objective of this trial is to evaluate the effectiveness of family-based MT targeting both adolescents and their parents to prevent adolescent substance use and enhance neurobehavioral self-regulation skills that play a major role in addiction development and mental health.

**Methods/design:**

The trial design is a superiority, two-arm, randomized controlled trial in which families will participate either in the full curriculum of the evidence-based Strengthening Families Program 10–14 (SFP 10–14, German adaptation) or in a mindfulness-enhanced version of this program (SFP-Mind). Both seven-session interventions are highly structured and will each be delivered over a period of approximately 7 weeks. The experimental intervention SFP-Mind is a modified version of the SFP 10–14 in which some elements were eliminated or changed to enable the inclusion of additional parent-directed and adolescent-directed mindfulness components. The primary outcome is adolescent self-reported alcohol use based on an alcohol initiation index at 18-month follow-up. Dispositional mindfulness, impulsivity, and emotion regulation will be included as secondary outcomes and potential mechanisms of action. The study will recruit and randomize 216 adolescents, aged 10–14 years, and their parents who will be followed up for 18 months.

**Discussion:**

This trial aims to evaluate the effectiveness of SFP-Mind for family-based prevention of substance use and promoting mental health in adolescence.

**Trial registration:**

German Register of Clinical Studies, DRKS00015678. Registered on 25 February 2019.

## Background

Substance use disorders (SUDs) are among the most frequent and costly mental disorders, both in the general population and among youth [1]. More than 15% of disability-adjusted life years (DALYs) worldwide are attributed to adolescents and emerging adults, with increased risks associated with all types of abusive substances [2]. Compared to the general population, the prevalence for SUDs is higher among young people in western cultures where excessive drinking and other substance abuse usually peaks in emerging adulthood [3, 4]. In Germany, as in most industrialized countries, alcohol is the primary substance abused by teenagers. Although youth drinking in Germany and Europe is moderately decreasing [5, 6], excessive drinking remains high compared to other regions in the world, such as the USA [7], and a substantial proportion of school-aged youths drink alcohol at levels associated with serious risk of harm [8]. Binge drinking directly leads to the placement of more than 23,000 German adolescents per year in emergency care (EC) for acute alcohol intoxication, with indirect substance-related EC cases far exceeding this number [9]. Other short-term and long-term consequences associated with the abuse of alcohol and other substances include serious injuries, violence, fatal road accidents, high-risk sexual activities, school drop-out, development of mental and addictive disorders, heart disease, and cancer as well as impairments of neurocognitive function and brain development [8].

Adolescence may be particularly important for preventive interventions because this period is accompanied by a rapid development of neurocognitive systems that promote novelty-seeking and sensation-seeking alongside risk-taking, with a growing interest in new exciting experiences, including the experimentation with substance use [10]. SUDs (and substance use) are typically initiated in adolescence [11]. Moreover, adolescence constitutes the final maturation phase before adulthood stability [12], and is characterized by heightened susceptibility to peer influences toward risk-taking behavior in this developmental stage [13]. Reducing the risk of SUDs and associated problems through effective interventions during the key developmental window of adolescence is a major public health priority [14].

Previous findings have demonstrated the importance of the family system as an early proximal context for youth development, and the family’s role in early-onset and accelerated problem substance use and related mental health problems. This research provides a strong rationale for prevention based on family-associated risk and resilience factors [15–17]. For example, parents influence their offspring’s substance use by their own substance use, by setting alcohol-related rules and norms, and by monitoring children’s whereabouts [18]. Moreover, parents contribute substantially to the development of self-regulation, psychological functioning, and adaptive strategies for managing negative emotions [19, 20]. Developmentally, the acquisition of self-regulatory competencies emerges from childhood onward; that is, during a time period in which parental influence is high and the social salience of peers is lower than in adolescence [21]. Supportive family relationships have proven themselves to be protective factors against the development of SUDs [22]. Parents with good socioemotional skills can effectively buffer vulnerability for mental disorders among their children, thus contributing to the development of resilience [23]. Programs aimed at promoting social–emotional skills in young adolescents are considered more effective when a carer is involved [15, 24]. Similarly, substance use prevention programs among children and adolescents, which are primarily school-based [14, 25], can be more effective when they also address family-based risk and resilience factors [15, 26].

The Strengthening Families Program for Parents and Youth 10–14 (SFP 10–14) [27] has established an evidence base for preventing the onset and escalation of adolescent substance use and other problem behavior [16, 17, 28–30]. Although several replication trials in European countries have not yielded the same desirable effects [31, 32], the SFP 10–14 is currently one of the most widely disseminated evidence-based prevention programs to promote individual (intrapersonal and interpersonal) competencies and familial resources for healthy and active development, and socioemotional resilience [33]. In our own randomized controlled trial (RCT), we found that the SFP 10–14 is feasible and effective in preventing tobacco use and conduct problems, especially in families with children who have an elevated psychosocial risk load [34, 35]. However, our study results, as well as the other replication trials [31, 32], suggest that program effects could be improved.

An emerging literature base suggests that mindfulness training (MT) may be a helpful addition to such programs as a strategy to target neurocognitive factors [36–40]. Mindfulness is commonly conceptualized within a framework of self-regulation and involves full attention to present-moment experience with an attitude of acceptance, non-judgment, and openness [41]. Clinical and neuroscientific research [36, 38, 42–49] indicates that mindfulness skills can be acquired by training, and that they are associated with improved cognitive flexibility and affective stability, including (inhibitory) control of attention, emotion regulation, and stress reactivity. Taken together, this research supports the notion that MT can foster improvements in top-down control and help individuals manage cognitions, feelings, and behaviors more effectively. These factors not only play a major role in repetitive addictive behaviors [12, 36–38, 40, 48, 50–52], but they also predict the initiation and onset of substance abuse problems and related risks [53, 54], as well as a range of other externalizing and internalizing psychiatric difficulties, which often precede SUD development [55–58].

The evidence base for versions of MT-based interventions is more established for adult than for adolescent populations. However, based on the positive results of a broad range of psychological and clinical outcomes (e.g., [48]), there is growing interest in the application of MT in youth populations in clinical and non-clinical settings [42, 43, 45, 46]. Although most of this research is thus far preliminary (more rigorous clinical trials are underway [59–61], existing studies overall indicate applicability, safety, and initial efficacy of developmentally appropriate versions of MT for children and adolescents. This generally supports the idea that MT can have a significant impact on health and resilience among children and adolescents.

Most existing MT-based prevention approaches were implemented in educational settings [42, 43] while rigorous prevention studies in other non-clinical settings, such as the family, are still lacking. However, initial results (two trials are currently underway [59, 60]) indicate that teaching parents to apply mindful practice in everyday interactions with their children (*mindful parenting*) can positively influence stress-sensitive cognitive and emotional processes relevant for emotional well-being within families, and healthy child development in families in general [62] as well as in families with adolescents exhibiting externalizing behaviors [63]. Coatsworth et al. [64, 65] integrated elements of mindful parenting into the SFP 10–14 (Mindfulness-enhanced Strengthening Families Program, MSFP). Their randomized controlled trial showed that teaching parents mindfulness in combination with parenting skills was superior to the standard SFP 10–14 in several domains of family functioning, in youth behavior management, and in parent well-being (effect sizes after 12 months: *d* = 0.19–0.46). The study indicates that integration of MT for parents into the SFP 10–14 is feasible and associated with enhanced program effects in family and parenting outcomes. However, the study does not include data on substance use and other child-level problem behavior-related outcomes; furthermore, MT was only delivered to the parents.

Since the development of self-regulation and other socioemotional competencies largely happens during the preadolescent period, a phase in which parental influence is still comparably strong, the delivery of MT to modulate risk factors of SUDs and associated psychopathology seems conceptually plausible. Given this and the promising initial results of child and adolescent-related MT approaches, we aim to integrate both child-directed MT and mindful parenting components into the proven structure of the SFP 10–14. We will encourage parents to support their adolescents in practicing mindfulness. We shall test the effectiveness of this mindfulness-augmented program in preventing adolescent alcohol use and improving self-regulation at 18-month follow-up as well as its impact on a spectrum of further individual and family-level outcomes such as parenting skills, child behavior, and family cohesion. We expect and hypothesize that adding mindfulness to the SFP 10–14 will increase the programs’ effectiveness on these outcomes.

## Methods/design

### Study design

We will apply a superiority, two-arm randomized, prospective, observer-blind controlled trial in which an MT-enhanced family-based prevention program will be compared with the SFP 10–14, a previously established efficacious “standard” program version without MT components. This program has been shown to improve symptoms of psychopathology and to delay uptake of substance use in previous studies [28–30], including a German adaptation trial [34]. Intervention conditions in both trial arms are comparable in the frequency and duration of personal contacts. Although inclusion of an additional no-treatment control group in a three-arm trial appeared a desirable option, we consider a two-arm trial adequate for two reasons: firstly, it is an adequate design to address the primary research question, whether MT adds value over the evidence-based standard program version (for a similar approach, see [59, 60, 65]); and, secondly, recruitment is often limited because program deliverers in the field are reluctant to refer families to RCT studies with non-active or minimal control group designs [66, 67].

### Participants and eligibility

Study participants will be children and adolescents aged 10–14 years and their parents or primary caregiver (one adolescent and one carer per family will be included in the analysis; the oldest adolescent aged between 10 and 14 years and the carer who attends the most sessions will be defined as index participants; in the case of equal participation of carers, inclusion will be decided by chance). We aim to recruit 216 families. Families will be excluded if the adolescent exhibits clinical symptoms of psychosis and/or suicidality and/or if family members’ limited knowledge of the German language compromises survey and group participation. The participation of the child or adolescent must be confirmed with written consent from all of their legal guardian(s) alongside that of the child or adolescent. The carer must confirm their own participation via written consent.

### Setting and recruitment

Recruitment shall take place at community-based child and family service institutions in the region of Hamburg, Germany, which will be chosen as recruiting centers if they reach a sufficient amount of families with their own qualified preventive service programs. As the SFP 10–14 is not routinely implemented in Hamburg, we will set up the required infrastructure for recruitment and program implementation in cooperation with and with support from the local authorities, as we have done for a similar study in the past [34]. Multiple recruitment strategies will be employed to reach a sufficient number of recruitment centers; recruitment includes distribution of written information and presentation of the program goals and study procedures by project representatives via local professional networks and specified agencies in the family health care and social service sector. Additionally, we will start initiatives to raise awareness in educational settings and at authority-level meetings.

**Fig. 1:**
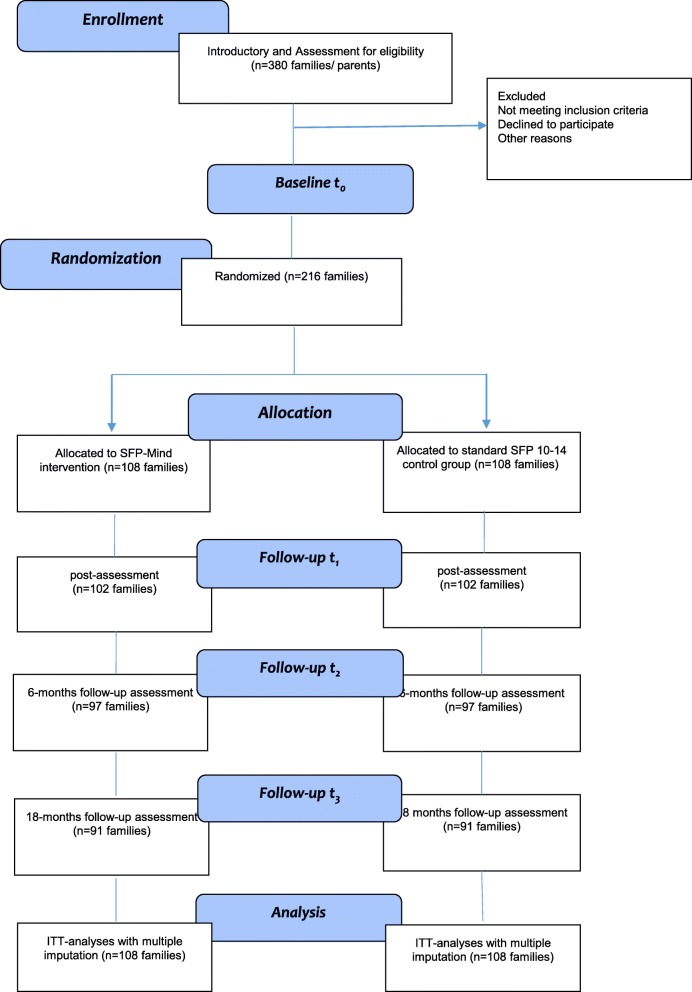
Consolidated Standards for Reporting Trials (CONSORT) diagram: participant flow, detailing recruitment, training, intervention, and assessment schedule. ITT intention-to-treat, SFP-Mind Strengthening Families with Mindfulness, SFP 10–14 Strengthening Families Program for Parents and Youth 10–14

Potential referrers will be informed about all aspects of the study and will agree to deliver both program versions in accordance with the study procedures, particularly those that concern randomization procedures, ethical standards, and qualified intervention delivery. Referring institutions will recruit potential participants and inform them about the prevention programs and the study procedures. Consent to participate in the family program (experimental or control) will take place prior to the baseline assessment. Interested families will be visited by a member of the research team for subsequent eligibility assessment and enrolment into the study. Written informed consent, with emphasis on withdrawal rights and ethical aspects as well as all relevant conditions of trial participation, will be obtained during the family visit. Baseline data will be collected before randomization. Trial participants are subsequently randomized to one of the two trial conditions. Recruitment/trial inclusion will be ongoing over a period of approximately 12 months. Figure 1 displays the anticipated CONSORT participant flow.

Program facilitators (experimental or control intervention) from cooperating institutions will take part in certified 3-day training (totaling approximately 24 h). Participating institutions shall receive a monetary compensation (€25 per intervention hour) from project funding. Participants in the trial will receive monetary compensation for their lost time for all assessments and at completion of the trial.

### Randomization procedure and blinding

Enrolled families will be randomized in a 1:1 allocation ratio into both intervention groups (SFP or SFP-Mind), as per a computer-generated randomization schedule stratified by recruitment center and intervention cycle. Multiple recruitment centers will be used for the trial. As soon as there are sufficient consenting participants for a single intervention cycle of seven sessions (typically six to eight families per intervention arm), the following random allocation process will be applied. First, an independent statistician will be informed by the study team about the sample size of participating families in the designed intervention cycle and create a concordant computer-generated randomization sequence, which will be concealed until assignment occurs. Second, a researcher independent to the study team will receive an anonymized index identification code for each family participating in that intervention cycle, including information on absolute time restrictions for participation (i.e., holidays, carers’ weekday absences), from the study team. The independent researcher will then randomly assign an index identification code to the randomized sequence. In the case that a defined time restriction of one family means incompatibility with the randomly allocated intervention timing, the family will be allocated to the other intervention arm by the independent researcher. This methodology is necessary to ensure family participation as some might have fixed time constraints to participate. In the event of reallocation due to individual time restrictions, the index identification code will be separately reported to the study team after the allocation process is finalized to allow for methodological scrutinization. See Additional file 2 for a flow chart of randomization procedures.

### Interventions

#### SFP 10–14 (control)

Families in the control group will participate in the standard SFP 10–14, which is a highly structured and evidence-based prevention program for families at the universal prevention level [27]. ›*Familien stärken*‹ is the certified German version, which relies on a video/DVD-based approach to learning the same as the original US program. In the narrated videos, participants see typical family interactions in diverse families and settings. To accommodate a contemporary urban setting and to increase authenticity and credibility, in extensive pilot work the original US-version video material was presented and discussed in focus groups of target families and translated as well as culturally adapted [68]. The program features seven weekly sessions of approximately 3 h. Per session, three group facilitators simultaneously work with 8–12 families, with parallel sessions for parents and adolescents during the first hour and sessions for the entire families during the second hour. All sessions include DVD presentations, role-plays, group discussion, input, social bonding, and skill-building activities. At the end of the program, a family meal is also provided to strengthen support and sharing between families. For more information on program content, see Bröning et al. [69].

#### SFP-Mind (experimental intervention)

The development of the experimental SFP-Mind intervention (›*Familien achtsam stärken*‹ [Strengthening Families with Mindfulness]) is part of the ongoing study. Implementation procedures and participant satisfaction were qualitatively assessed in a pilot intervention based on a small sample of target families (*N* = 5). SFP-Mind will integrate intrapersonal and interpersonal mindfulness practice into the evidence-based SFP 10–14 while maintaining the validated structure of the original program.

We focus on teaching mindfulness to adolescents to target the neurobehavioral, self-regulatory mechanisms implicated in developmental pathways of addictive behavior (such as reward and response inhibition, impulsivity, and emotion processing) and to strengthen developmental competencies. But the novel program also provides mindfulness modules for parents to support their own and adolescent mindfulness activities outside the regular weekly group sessions, which are derived from programs of mindful parenting [62–65].

This approach targets the family as a system and reflects a developmental perspective in which parents are considered influential agents for adolescent development. We assume that mindfulness holds particular promise because it can help manage instability of intrapersonal and interpersonal affective states (e.g., stress, anger, negative mood), which commonly exists at the family level in this developmental period, and often predicts substance use [26, 70]. The newly added mindfulness program modules (e.g., focused attention, deep breathing, cultivating a kind attitude toward self and others, setting positive intentions) draw on youth-specific adaptations of mindfulness-based interventions (such as the 8-week curriculum of mindfulness-based cognitive therapy, MBCT), but will be relatively brief compared to other formal mindfulness interventions, and will be designed to reflect age-related developmental needs and characteristics of youth 10–14 years old (e.g., with regard to attention span, cognitive capacities, language, physicality, relevant content, time involved in the intervention, and home practice in a situation with competing time demands). The selection of age-appropriate mindfulness modules for our targeted population builds on the current knowledge base of mindfulness training in non-clinical youth populations, which mainly derives from established school-based approaches [71], such as the “Learning to Breathe” manual [72, 73] and other programs [74, 75].

The new mindfulness components will be integrated into the parallel adolescent-directed, parent-directed, and family-directed sessions in the seven consecutive sessions with emphasis on one thematic key aspect of mindfulness per week. The seven thematic key aspects will be Breath, Attention/Awareness, Body/Stress, Feelings/Emotions, Thoughts, Compassion/Happiness, and Mindfulness in Everyday Living. Consideration of youth-specific needs for experiential, interactive, and skill-oriented learning are the guiding principles in the development and integration of the mindfulness modules.

One goal is to stimulate mindfulness practice outside the weekly program sessions. Participants will thus be strongly encouraged to practice meditation and MT on a daily basis and to protocol their practice on diary cards. Parents will be instructed to support adolescent home practice. Moreover, home practice MT-related content, such as guided meditations, will be provided in written form as well as in audio files and a computer/smartphone app. This material will be specifically developed for study purposes and serves as a practical and user-friendly method to foster home practice of program content. Importantly, the app is therefore not designed to collect data (however, frequency of use of app content will be analyzed). Use of the app is voluntary, as not every child may have access to a tablet or smartphone, and will be fully compatible with German regulations of data security. Existing integrations of (parent-directed) mindfulness training into the SFP 10–14 [64, 65] indicate that facilitating mindfulness practice via didactic presentations about principles of mindfulness, modeling of mindfulness practices, and group interactive discussions are feasible formats to accommodate a family-based substance use prevention setting. Additional file 1 provides a schematic overview of the experimental and control interventions.

### Intervention fidelity

To ensure both programs are delivered with expertise and fidelity, at least two of the three intervention trainers will be social workers from the field with experience in working with adolescents or families, who have completed the mandatory 3-day certified SFP 10–14 training program. The SFP-Mind facilitators will either be the program developers or facilitators trained by experienced personnel in teaching the novel mindfulness modules and require having personal experience with MT prior to starting the group trainings. This way, we aim for an adequate standard for MT delivery that is comparable to other mostly school-based MT approaches among universal youth populations [43, 71–74] and family-based approaches [65]. While we acknowledge the importance of mindfulness skills among trainers, our approach reflects externally valid conditions which might be found in real-world conditions, thus maximizing generalizability. Scripted manuals in both conditions support clarity, fidelity, and adherence, which will be monitored during the study. Specifically, two independent raters will estimate adherence in videotapes of selected sessions using a list of prescribed activities as applied in a previous study [34] and with modifications made for novel MT program content. Data on acceptability of the programs by the study participants and the facilitators will also be collected.

### Baseline assessments and follow-ups

**Fig. 2:**
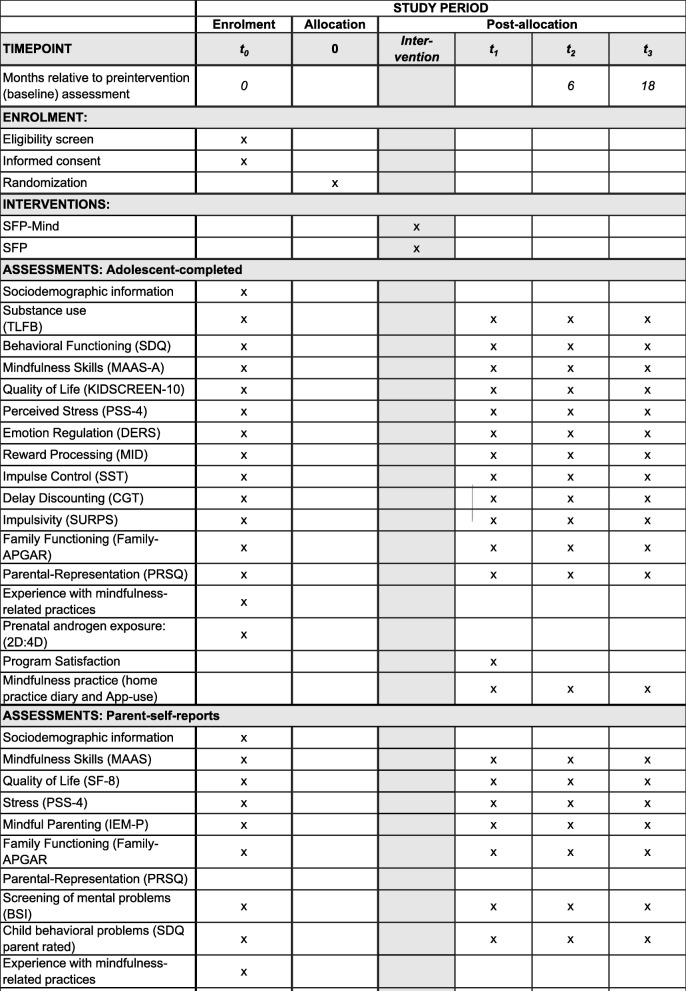
Standard Protocol Items: Recommendations for Interventional Trials (SPIRIT) diagram detailing trial activities and measures and their timing. 2D:4D second and fourth-finger lengths ratio, BSI Brief Symptom Inventory, CGT Cambridge Gambling Task, DERS Difficulties in Emotion Regulation Scale, IEM-P Interpersonal Mindfulness in Parenting scale, MAAS Mindful Awareness and Attention Scale, MBI-TAC Mindfulness-Based Interventions—Teaching Assessment Criteria, MID Monetary Incentive Delay, PRSQ Parental-Representation-Screening-Questionnaire, PSS-4 Perceived Stress Scale German Version, SDQ Strengths and Difficulties Questionnaire, SF-8 Short-Form Health Survey, SFP Strengthening Families Program, SFP-Mind Strengthening Families with Mindfulness, SST Stop-Signal Task, SURPS Substance Use Risk Profile Scale, TLFB Timeline Follow-Back

Study outcomes for adolescents and their parents/carers will be assessed by trained research staff blinded to the intervention condition at the families’ homes. Participants will be surveyed at baseline (prior to randomization), post intervention (approximately 8 weeks after baseline), as well as 6 months and 18 months (follow-ups) after post assessment. Fidelity and adherence data (i.e., ratings of training sessions) will be gathered during the intervention period (7 weeks). Figure 2 shows an overview of measures from study participants at each time point.

### Outcome measures

Most study outcome measures, including the data on substance use, are self-report questionnaire data, which will be completed either on paper or via tablets. Substance use data will not be biochemically validated. In our previous study [34] we verified the adolescents’ self-report data by analyzing their urine samples for cotinine (threshold 50 ng/ml), ethyl glucuronide (ETG; threshold 0.1 mg/l), and tetrahydrocanabinol carboxylic acid (THC carboxylic acid; threshold 10 ng/ml). We found that the level of underreporting was very low (0.4–14% for various substances) and therefore we generally expect valid self-report data on substance use in the forthcoming trial. Secondary outcomes include questionnaire data and experimental data measured with a comprehensive (neuro)behavioral test battery (see later). Details of the measures and the time points at which they will be collected are shown in Fig. 2 (also see Additional file 3 for the filled SPIRIT 2013 Checklist).

### Primary outcomes (adolescent-completed)

The primary trial outcome to estimate the effectiveness of the novel MT-extended prevention program will be adolescent drinking 18 months after the intervention (one adolescent per family will be included in the analysis). While other substances are also relevant, we consider a primary focus on alcohol to be adequate particularly because: Germany is a high-consumption country, with alcohol being the primary substance of abuse [5]; delay of alcohol use is considered a primary substance use prevention goal, since development of SUD has repeatedly been shown to be predicted by the age of drinking onset [13, 76, 77]; significant proportions of youth in Europe start drinking at levels that is harmful to their healthy development early in their lives [78]; and addictive developmental trajectories are typically characterized by early onset and rapid acceleration of drinking [79]. To assess drinking we will form an alcohol initiation index (AII) that consists of the following items: “Have you ever tried to drink alcohol (just a sip but not a full drink)?”; “Have you ever had a full drink of alcohol (more than just a few sips)?”; “Have you felt (minimal) drunk?”; and “Have you ever felt (severely) drunk?” All four items will be answered according to a binary yes–no format and coded as 1 for yes and 0 for no. Comparable index-based measures for lifetime substance use were used extensively in previous studies [80–82].

Accordingly, we will examine the four lifetime use items individually, but the primary outcome will be the sum of the scores of these lifetime use items at the 18-month follow-up. Inconsistencies in the reports of lifetime alcohol use will be corrected. In cases where a participant reports a lifetime use behavior at one data collection point but reports no such use at a later collection point, the later report will be corrected to reflect the previously reported initiation of that behavior. For analytic purposes, lifetime use measures will be adjusted to control for baseline use. These adjusted lifetime use measures, called new-user rates, indicate whether use was initiated since baseline. Prior studies have reported the validity of similar substance use indices [82, 83].

### Secondary outcomes (adolescent and parent-completed)

Additional drinking outcomes, such as frequency and quantity of alcohol and drug use other than alcohol within the past 30 days prior to assessment, will be included as secondary outcomes and assessed using the standardized, calendar-based Timeline Follow-Back (TLFB) interview format [84]. As MT can be expected to foster improvements in top-down control [38, 41–44], we focus on experimental measures of self-regulation as key secondary outcomes in this study. Although self-regulation is a multidimensional “umbrella construct” across different scientific disciplines [85], p. 2693, definitions typically include the inability to delay gratification, insensitivity to negative consequences, distractibility, and the inability to inhibit or control impulsive behavior [85–87]. To specifically assess self-regulation in this study, we use versions of the Cambridge Gambling Task (CGT) from the Cambridge Cognition Neuropsychological Test Automated Battery (CANTAB; Cambridge Cognition) as a measure of delay discounting [53], the Monetary Incentive Delay (MID) reaction time task to assess reward sensitivity [88], and the Stop-Signal Task (SST) that is a widely used index of impulse control [89, 90]. All of these tasks have been used in previous studies [53, 54] and will be adapted to the specific needs of the present trial.

Moreover, we will include a range of individual and family-level variables as secondary outcomes as indicators for risk or resilience in the developmental course of SUD and related difficulties. These outcomes (A indicates outcome is adolescent-directed, P indicates it is parent-directed) are as follows:

- Social, emotional and behavioral functioning (SDQ—Strengths and Difficulties Questionnaire German youth version) [91] (A + P)
- Screening of mental problems (BSI—Brief Symptom Inventory) [92] (P)
- Mindfulness Skills (MAAS—Mindful Awareness and Attention Scale) [93, 94] (A + P)
- Health-related quality of life (KIDSCREEN-10) [95] (A)
- Short-Form Health Survey (SF-8) [96] (P)
- Stress (PSS-4—Perceived Stress Scale German Version) [97] (A + P)
- Emotion regulation (DERS—Difficulties in Emotion Regulation Scale) [98, 99] (A)
- Reward processing (Monetary Incentive Delay Task) [88] (A)
- Impulse control (Stop Signal Task) [89, 90] (A)
- Delay discounting (Cambridge Gambling Task) (CANTAB; Cambridge Cognition) (A)
- Impulsivity (SURPS—Substance Use Risk Profile Scale) [100] (A)
- Mindful parenting (IEM-P—Interpersonal Mindfulness in Parenting scale) [62] (P)
- Family functioning (Family APGAR) [101] (A + P)
- Parental-Representation-Screening-Questionnaire (PRSQ) [102] (A + P)
- Previous experience with mindfulness-related practices (e.g., meditation, yoga, etc.; purpose designed) (A + P)
- Prenatal androgen exposure: the second and fourth-finger lengths ratio (2D:4D) will be scanned and used as a proxy of intrauterine exposure to gonadal steroids, which is a covariate for addiction development [103] (A)

### Implementation outcomes

For the participants in the SFP-Mind group only, we will assess mindfulness practice using several questions about sustained use of mindfulness practice in the follow-up assessments. In both intervention groups, we will also assess the following variables:

- Program satisfaction: participants (parents and adolescents) will rate how satisfied they are with the program based on the Treatment Satisfaction Questionnaire (*Fragebogen zur Beurteilung der Behandlung*, FBB) [104] (A + P)
- Trainer adherence to principles and concepts of mindfulness will be rated according to a purpose-designed rating scale based on the MBI:TAC (Mindfulness-Based Interventions—Teaching Assessment Criteria) proposed by Crane et al. [105]
- Ongoing mindfulness practice (formal (e.g., meditation of deep breathing) and informal mindfulness practices; purpose designed; analysis of home practice diary and evaluation of frequency of app use) (A + P)

### Sample size

Development of the intervention is part of the ongoing study; thus, the empirical base for calculation of expected intervention effects is limited at the outset. However, the power calculation was based on our prior SFP 10–14 evaluation [34] and current evidence for MT in youth [42, 43]. Based on this research, we expect a small to medium effect size (ES) of *d* = 0.40 for the primary endpoint at 18-month follow-up in favor of SFP-Mind over standard SFP in a two-group analysis of covariance (ANCOVA). A sample size of *n*_1_ = *n*_2_ = 91 (total *n* = 182 families) is sufficient to detect effects of this size with a statistical power of 80%, a two-sided α level of 5% (estimated with PASS 15; NCSS, LLC, Kaysville, UT, USA) (ANCOVA), and an assumed correlation of *r* = 0.30 between the baseline and the follow-up measurements. Based on our previous SFP 10–14 trial [34] we expect a loss to follow-up (after 18 months) of 15%, and will therefore oversample by this percentage (total *n* = 216 families).

### Analysis plan

Analyses are planned and supervised by the trial statistician (AD). Study eligibility and enrollment will be summarized according to the CONSORT statement [106]. Descriptive statistics of baseline variables will be assessed by intervention arm. Analyses will be based on the intention-to-treat population, thus including data from all participants who provide baseline data and are subsequently randomized to one of the two trial conditions. Missing follow-up data will be imputed using multiple imputation methods. For the primary analysis, a mixed, linear, repeated-measurement ANCOVA will be conducted, with alcohol initiation (in the form of an initiation index) as the dependent variable; group, time, and interaction between group and time and recruitment center as fixed effects; baseline scores of alcohol use as covariates; adolescent and intervention groups within the study arm as random effects; and time as a repeated effect. The contrast between both groups at the 18-month follow-up will be assessed in a confirmatory manner. We will report adjusted group differences with corresponding 95% confidence intervals (CIs), *p* values, and Cohen’s *d* effect sizes. A systematic examination of factors associated with loss to follow-up will be conducted. The two-sided type I error will be set at 5%. An additional analysis will be conducted on a per-protocol data set as sensitivity analysis. A further sensitivity analysis will be carried out using the data set with all available cases (without any imputation of missing data). The secondary outcomes as well as a potential moderating role of individual and family-level variables and adherence to the intervention and engagement in home practice will be examined in an exploratory manner with appropriate methods. The other model parameters will be set as in the primary outcome analysis. The safety endpoints will be determined using frequency tables and, if possible, using mixed logistic regressions to compare the event frequencies. Interim analyses are not planned. A detailed statistical analyses plan will be prepared and finalized before the code is broken. Statistical analyses will be carried out with SPSS Version 23 or newer (IBM Corp., Armonk, NY, USA).

Mechanisms of action and subgroup analyses will be identified within the wider consortium (see “Trial governance”) using structural equation models with Mplus Version 5 or newer [107]. These methods require larger data sets [108]. Therefore, we will employ standardized measures for central variables across the consortium and collapse data sets from five clinical trials on mindfulness-oriented interventions in different populations for sufficient statistical power. We will explore potential mediators of intervention effects with a focus on experimental measurements of self-regulation (i.e., Monetary Incentive Delay Task, Stop Signal Task, Cambridge Gambling Task), mindfulness skills (i.e., Mindful Awareness and Attention Scale, MAAS), and emotion regulation (i.e., Difficulties in Emotion Regulation Scale, DERS).

### Methods against bias

We take the following action to minimize potentials for biased results. Assessments will be conducted by trained interviewers unaware of the study condition, at home and individually in separate rooms for participating family members to ensure privacy and confidentiality and to reduce drop-out. Blinding of families and intervention facilitators is not possible. To maintain program retention, during the family sessions we offer child care for families who have younger siblings they cannot leave at home alone [69]. Moreover, participating families will be financially rewarded for complete program retention and for each completed follow-up data collection. To avoid crossover in the intervention groups, we aim for implementation of parallel group training with regard to both intervention conditions.

Data management will be performed locally following the guidelines of Good Clinical Practice (GCP) and will be monitored (including source data verification), collected, and preprocessed by the Clinical Trial Center North, thus an external clinical research organization (CRO).

A Data and Safety Monitoring Board (DSMB) will be implemented once the recruitment has started with regular meetings (preferably by telephone conferences), with an open and closed part with and without the coordinating investigator. The DSMB will monitor study and recruitment progress, protocol deviations, loss-to-follow-up data, serious adverse events (SAE) and adverse events (AE), and all problems of the study and decide independently from the coordinating investigator whether the study can be continued or a recommendation is given to change the design or stop the trial. The members of the DSMB will be experienced scientists who are not otherwise involved in the study, independent of the investigators and the trial sponsor, and will have no conflict of interests.

Recruitment centers and all researchers conducting the consenting process and baseline assessments will be repetitively trained to conceal any information which could lead the participants to associate an intervention timing with an intervention arm in order to improve allocation concealment.

The Institute of Medical Biometry and Epidemiology at the University Medical Center Hamburg–Eppendorf supports the trial in terms of database development, randomization, data management, and analyses (including for plausibility check and analysis of the missing data structure). It will also provide the DSMB with safety reports and methodological expertise if required.

Prepublication of the trial design and trial registration in a public database before recruitment has started, and adherence to the CONSORT statement for randomized controlled trials will further minimize potentials for bias. Data collection and entry will be conducted according to standardized protocols. Self-report data will be collected mainly using digital methods, thus minimizing errors of data entry. Any data collection using paper–pencil measures (e.g., assessment of safety endpoints) will be computerized by trained staff using the EpiData software, with double entry and range checks for data values. All data will be stored securely and in line with current guidelines and regulations concerning data safety and confidentiality of the study participants.

Trial results will be reported at international conferences and meetings and published in open access and peer-review journals to ensure a maximized and high-quality dissemination of the study findings.

### Trial governance

The study is part of the national study group “IMAC-Mind” (Improving Mental Health and Reducing Addiction in Childhood and Adolescence through Mindfulness: Mechanisms, Prevention and Treatment), which focuses on the clinical utility of mindfulness-oriented approaches to prevention and treatment of SUD in different developing populations, from the prenatal period throughout the second decade of life. The consortium comprises eight subprojects with five clinical studies of mindfulness-based approaches to SUD prevention and treatment (see www.imac-mind.de) and is coordinated by the first and the last authors. It is supervised by a project steering committee comprising the principal investigators of all subprojects. The trial is supported by the Institute of Research and Education GmbH associated with the Medical School Hamburg (MSH). It is monitored by an external CRO and overseen by an independent DSMB. The project receives national funding by the German Federal Ministry of Education and Research (Grant: 01GL1745F). The sponsor of the trial described in this protocol is the University Medical Center Hamburg–Eppendorf. The funding source has no role in the design of this study and will not have any role during its execution, analyses, interpretation of the data, or decision to submit results.

### Ethics

We will design, conduct, and evaluate the trial following the rules of GCP and commit ourselves to the Declaration of Helsinki [109]. Names and person-related data will be treated according to the conditions of the German Data Protection Act. Informed consent will be obtained from all participants prior to participation and can be withdrawn at any time.

Although the planned prevention trial targets universal families, it may assemble families with children manifesting behavioral difficulties who may be more willing to apply to participate in the study and/or may be prioritized in practitioner referrals as families in need of support. Risks associated with trial participation may thus include harmful peer modeling effects which may arise from aggregating youth with early indications of mental health problems. Participant information and safety will therefore be ensured by careful monitoring and supervision of intervention delivery personnel, documentation, and stopping and referral rules where appropriate. Beyond this issue, prior research on comparable interventions indicates no harm associated with trial participation.

SAE and AE will be carefully monitored, documented, and reported to the coordinating investigator and the Data and Safety Monitoring Board (DSMB) members within 1 week of the initial observation to determine the benefit–risk of trial continuation and adequate participant support.

The study was granted ethical approval by the responsible Ethics Committees of the Chamber of Physicians Hamburg (PV5932) and the study was registered in the DRKS public database (DRKS00015678). Any changes to this study protocol will be reported to the Ethical Committee for further review and modifications will be subsequently made in the trial registry if necessary.

## Discussion

This trial builds on the emerging evidence for the preventative utility of MT in youth and family settings, and aims to evaluate the effectiveness of a mindfulness-enhanced family-based substance use prevention program targeting both adolescents and their parents to prevent adolescent substance use using randomized controlled methods in a comparably large sample and a substantial follow-up period. Existing studies of MT among young people are largely limited to educational settings, and there are no controlled studies on both parent-directed and adolescent-directed approaches. Moreover, it is unclear whether such MT has preventive effects for substance use outcomes in adolescence.

In this trial, we integrate MT into an established prevention program that has a sound evidence base as a successful program to prevent adolescent substance use and promote positive youth development. Testing MT against an already efficacious program in an active control group design provides a direct estimate of the additive effects of MT over a program that is considered the current best practice for family-based substance use prevention [33]. While MT is usually delivered as a standalone intervention, we will directly test the assumption that an effective program can be improved using MT. By integrating the family as an important socialization context, we aim to contribute to the evidence base of MT approaches outside the educational setting, where youth-directed MT approaches are typically implemented. Finally, while we rely on self-report data for the primary substance use outcome, we include neurobehavioral measures to explore effects of MT on self-regulation skills implicated in addiction development and mental health.

With this trial, we expect to contribute to establishing MT as an effective approach for family-based prevention of substance use and promoting mental health in adolescence and to provide new insights into the mechanisms of action of MT.

### Trial status

The trial is ongoing. Recruitment of participants started in December 2019 and is expected to be completed in December 2020 (at the earliest). Protocol version #2; protocol version date January 8, 2020.

## Supplementary information

**Additional file 1.** Schematic overview of the experimental and the control intervention.

**Additional file 2.** Randomization procedures.

**Additional file 3.** SPIRIT 2013 Checklist: Recommended items to address in a clinical trial protocol and related documents.

## Data Availability

Study material (information sheets) is available to the public and can be found at the websites http://www.familien-staerken.info and https://imac-mind.de or by request. Anonymized study data and statistical codes to analyses may be made available on request following study closure.

## Abbreviations

2D:4D: Second and fourth-finger lengths ratio
AE: Adverse events
ANCOVA: Analysis of covariance
BSI: Brief Symptom Inventory
CANTAB: Cambridge Cognition Neuropsychological Test Automated Battery
CGT: Cambridge Gambling Task
CI: Confidence interval
CONSORT: Consolidated Standards for Reporting Trials
CRO: Clinical research organization
DALY: Disability-adjusted life year
DERS: Difficulties in Emotion Regulation Scale
DRKS: Deutsches Register Klinischer Studien [German Clinical Trials Register]
DSMB: Data and Safety Monitoring Board
DVD: Digital versatile disc
EC: Emergency care
ES: Effect size
ETG: Ethyl glucuronide
GCP: Good Clinical Practice
GmbH: Gesellschaft mit beschränkter Haftung [limited liability company]
IEM-P: Interpersonal Mindfulness in Parenting scale
IMAC-Mind: Improving Mental Health and Reducing Addiction in Childhood and Adolescence through Mindfulness: Mechanisms, Prevention and Treatment
MAAS: Mindful Awareness and Attention Scale
MBCT: Mindfulness-based cognitive therapy
MBI-TAC: Mindfulness-Based Interventions—Teaching Assessment Criteria
MID: Monetary Incentive Delay
MSFP: Mindfulness-enhanced Strengthening Families Program
MSH: Medical School Hamburg
MT: Mindfulness training
PRSQ: Parental-Representation-Screening-Questionnaire
PSS-4: Perceived Stress Scale
RCT: Randomized controlled trial
SAE: Serious adverse events
SDQ: Strengths and Difficulties Questionnaire
SF-8: Short-Form Health Survey
SFP 10–14: Strengthening Families Program for Parents and Youth 10–14
SFP-Mind: Strengthening Families with Mindfulness
SST: Stop-Signal Task
SUD: Substance use disorder
SURPS: Substance Use Risk Profile Scale
THC: Tetrahydrocanabinol
TLFB: Timeline Follow-Back
