# Supplementary figures and images for: Does a mindfulness-augmented version of the German Strengthening Families Program reduce substance use in adolescents? Study protocol for a randomized controlled trial
Source: Trials. 2020 Jan 28;21:114. doi: 10.1186/s13063-020-4065-1 (PMC6988370; doi:10.1186/s13063-020-4065-1)

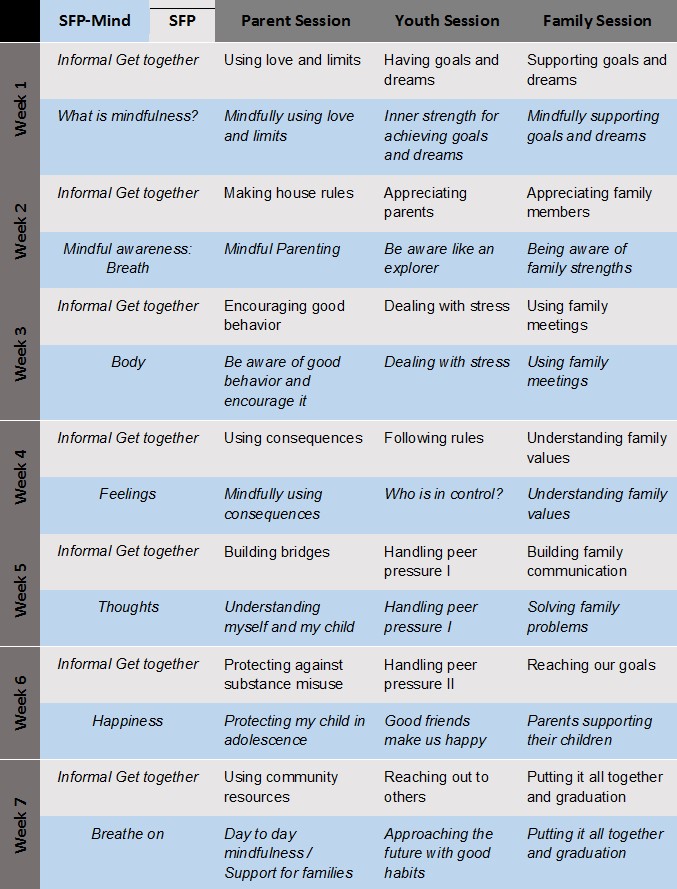

Supplement: Supplementary file 1 — Additional file 1. Schematic overview of the experimental and the control intervention. [file 13063_2020_4065_MOESM1_ESM.jpg]
