# Supplementary material for: Does a mindfulness-augmented version of the German Strengthening Families Program reduce substance use in adolescents? Study protocol for a randomized controlled trial
Source: Trials. 2020 Jan 28;21:114. doi: 10.1186/s13063-020-4065-1 (PMC6988370; doi:10.1186/s13063-020-4065-1)
Supplement: Supplementary file 2 — Additional file 2. Randomization procedures. [file 13063_2020_4065_MOESM2_ESM.pptx]

## Slide 1
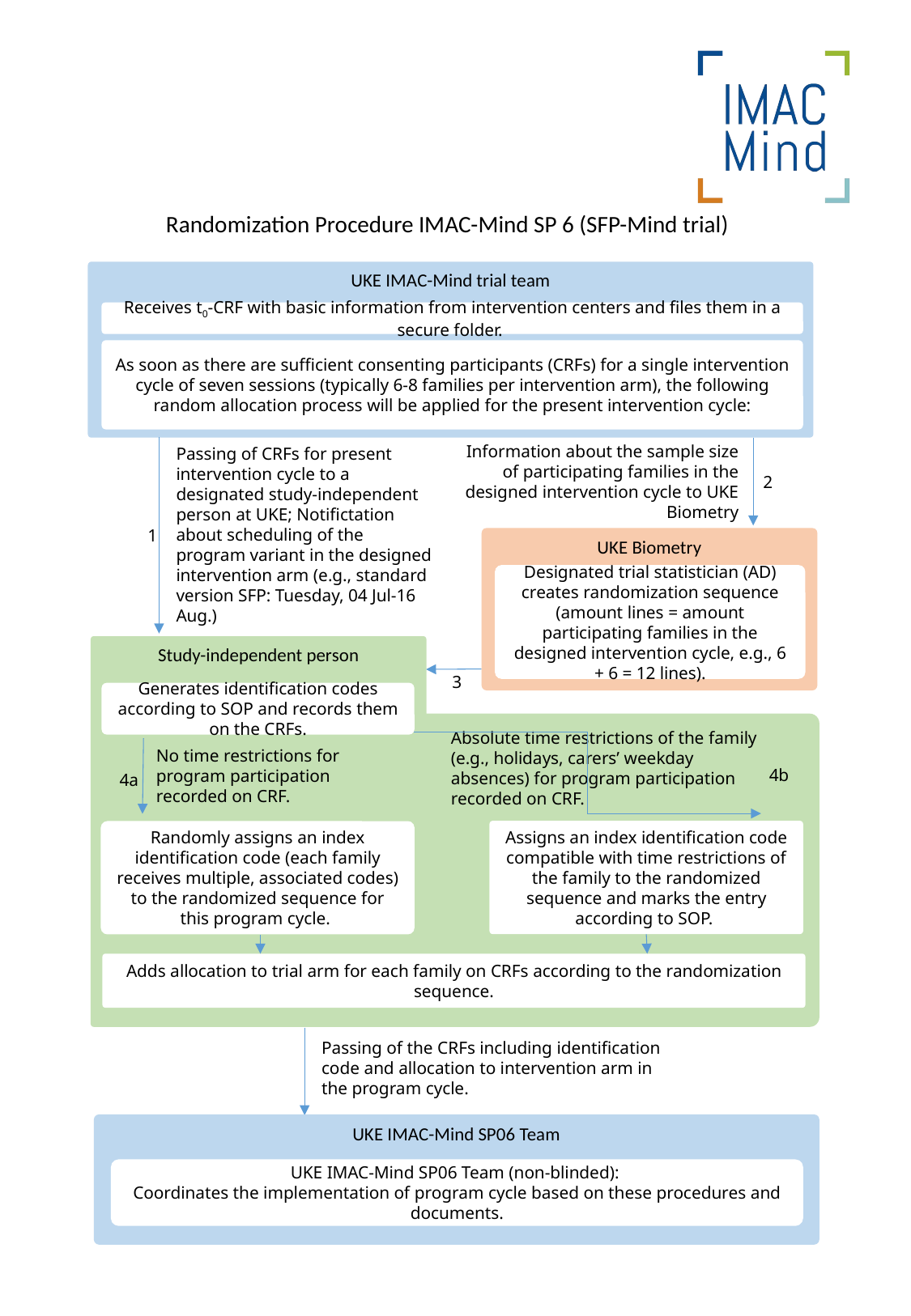

Randomization Procedure IMAC-Mind SP 6 (SFP-Mind trial)
UKE IMAC-Mind trial team
Receives t0-CRF with basic information from intervention centers and files them in a secure folder.
As soon as there are sufficient consenting participants (CRFs) for a single intervention cycle of seven sessions (typically 6-8 families per intervention arm), the following random allocation process will be applied for the present intervention cycle:
Information about the sample size of participating families in the designed intervention cycle to UKE Biometry
Passing of CRFs for present intervention cycle to a designated study-independent person at UKE; Notifictation about scheduling of the program variant in the designed intervention arm (e.g., standard version SFP: Tuesday, 04 Jul-16 Aug.)
2
1
UKE Biometry
Designated trial statistician (AD) creates randomization sequence (amount lines = amount participating families in the designed intervention cycle, e.g., 6 + 6 = 12 lines).
Study-independent person
3
Generates identification codes according to SOP and records them on the CRFs.
Absolute time restrictions of the family (e.g., holidays, carers’ weekday absences) for program participation recorded on CRF.
No time restrictions for program participation recorded on CRF.
4b
4a
Assigns an index identification code compatible with time restrictions of the family to the randomized sequence and marks the entry according to SOP.
Randomly assigns an index identification code (each family receives multiple, associated codes) to the randomized sequence for this program cycle.
Adds allocation to trial arm for each family on CRFs according to the randomization sequence.
Passing of the CRFs including identification code and allocation to intervention arm in the program cycle.
UKE IMAC-Mind SP06 Team
UKE IMAC-Mind SP06 Team (non-blinded):
Coordinates the implementation of program cycle based on these procedures and documents.
